# Rapid Response Teams in low and middle-income countries: a scoping review

**DOI:** 10.62675/2965-2774.20250155

**Published:** 2025-10-01

**Authors:** Larissa Bianchini, Luiz Marcelo Almeida de Araújo, Daryl Jones, Bruno Adler Maccagnan Pinheiro Besen

**Affiliations:** 1 Department of Internal Medicine Faculdade de Medicina Universidade de São Paulo São Paulo SP Brazil Postgraduate Program in Medical Sciences, Department of Internal Medicine, Faculdade de Medicina, Universidade de São Paulo - São Paulo (SP), Brazil.; 2 Hospital das Clínicas Faculdade de Medicina Universidade de São Paulo São Paulo SP Brazil Hospital das Clínicas, Faculdade de Medicina, Universidade de São Paulo - São Paulo (SP), Brazil.; 3 Instituto de Pesquisa HCor São Paulo SP Brazil Instituto de Pesquisa HCor - São Paulo (SP), Brazil.; 4 Department of Intensive Care Austin Hospital Melbourne Victoria Australia Department of Intensive Care, Austin Hospital - Melbourne, Victoria, Australia.; 5 Instituto D’Or de Pesquisa e Ensino São Paulo SP Brazil Instituto D’Or de Pesquisa e Ensino - São Paulo (SP), Brazil.

**Keywords:** Developing countries, Hospital rapid response team, Goals, Incidence, Critical illness, Patient reported outcome measures, Patient care planning, Cardiopulmonary resuscitation, Intensive care units, Brazil

## Abstract

**Background:**

Rapid Response Teams have been widely implemented in high-income countries and play a crucial role in the early identification and management of clinically deteriorating patients. However, their implementation in low and middle-income settings has not been adequately described. Our goal was to map the current evidence in this setting.

**Methods:**

We conducted a scoping review to map the published literature about Rapid Response Teams in low- and middle-income countries, according to year of publication, study type, team composition, reported outcomes, and potential roles of the team.

**Results:**

After screening 6,679 studies, 52 fulfilled eligibility criteria: 36 full-text studies and 16 conference abstracts. Most of the studies were from Brazil (51.2%), followed by India (19.2%) and Turkey (7.7%), with the two earliest reports being conference abstracts published in 2009. The predominant design was before-and-after studies (20; 38.4%), followed by cohort studies (16; 30.8%). An intensive care unit physician was always a member of the Rapid Response Teams in 55.9% of the studies and an intensive care unit nurse in 23.5%. The number of Rapid Response Teams calls in the before-and-after studies ranged from 2.39 to 124 per 1,000 admissions. Reported outcomes varied, with most studies focusing on mortality (26, 50%) and code blue incidence (21; 40.4%). Four (7.7%) studies reported an active role of Rapid Response Teams in goals of care discussions.

**Conclusion:**

We found that evidence on Rapid Response Teams in low- and middle-income countries remains limited, with a time lag in publications compared to high-income countries. Our findings highlight the need for further studies and policy initiatives to evaluate the effectiveness of implementing Rapid Response Teams in resource-constrained settings.

## INTRODUCTION

**Figure 1:**
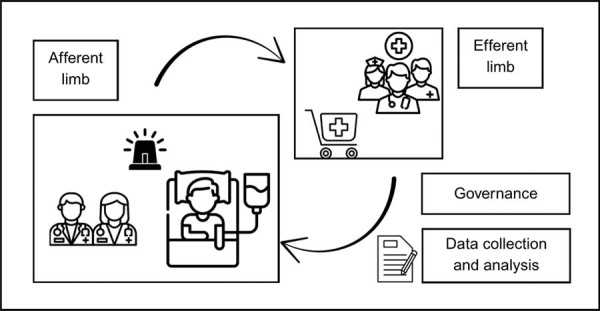
Components of the Rapid Response System. The Rapid Response System consists of four key components: the afferent limb, responsible for detecting clinical deterioration and triggering the response; the efferent limb, which includes the Rapid Response Team; the quality improvement limb, focused on data collection and feedback; and the administrative limb, which oversees coordination and system governance.

Rapid Response Teams (RRTs) are expert teams that review patients with signs of objective clinical deterioration outside of the intensive care unit (ICU).^( 1 )^Implementing RRTs has been associated with decreased intra-hospital cardiac arrests and mortality outside the ICU.^( 2 - 5 )^ Quality commissions established that the presence of RRT is a parameter of good hospital care.^( 6 , 7 )^ A mature Rapid Response System (RRS) identifies respiratory distress, sepsis, altered consciousness, and further organ dysfunction in the wards.^( 8 , 9 )^ The main components of the RRS include the afferent limb, efferent limb, administrative structure, and data collection for quality improvement, as illustrated in figure 1 . Furthermore, RRT calls can sometimes trigger goals of care discussions and do-not-resuscitate decisions.^( 10 , 11 )^Composition of the team is heterogeneous in the literature, with models varying from nurse-lead in England and the United States to an intensive care doctor as the team leader in Australia, referred to as Medical Emergency Team (MET).^( 1 )^ Critical care outreach teams (CCOT) usually consist of specialized nurses who, besides attending RRT calls, follow patients after ICU discharge and reevaluate patients with multiple RRT activations.^( 12 )^

Although RRT, MET, and CCOT are well established in high-income countries (HICs), data from low- and middle-income countries (LMICs) are less reported. Up to 96% of the countries included in published systematic reviews are HICs from Europe, North America, and Oceania,^( 5 , 9 , 13 )^even though the burden of critical illness in LMICs may comprise up to 85% of critically ill patients worldwide. There is a shortage of trained staff, research funding, and cultural barriers that decelerate growth and may prevent adequate implementation of initiatives as RRTs in constrained settings.^( 14 )^ However, in these resource-constrained settings, the benefits of RRTs on hospital outcomes could be even higher. In such settings, the RRTs might evolve to perform as a true “ICU without borders”,^( 15 )^ as organ support provision in the wards for some days may occur because of the scarcity of ICU beds.^( 16 )^ Even though RRTs may be an intuitive solution for management of critically ill patients in LMICs, whether there are consolidated teams in LMICs and if the potential benefit described for HICs on hospital mortality and cardiac arrest sustains in LMICs is underreported.

To address this question, we conducted a scoping review to map the published literature about RRT in LMICs and analyze knowledge gaps, focusing on team composition, potential roles of the RRT, and impact on hospital outcomes.

## METHODS

### Study design

We performed a scoping review to identify the published literature about RRT in LMICs, adhering to the Preferred Reporting Items for Systematic reviews and Meta-Analyses Extension for Scoping Reviews (PRISMA-Scr).^( 17 )^We followed the Joanna Briggs Institute (JBI) Manual for Evidence Synthesis to develop the study protocol and registered the protocol in the Open Science Framework (https://osf.io/3zsae/). The study question was structured using the acronym “PCC” as follows: “Among hospitalized patients with clinical deterioration (population), what is the role of MET / RRT / CCOT (concept) in low and middle-income countries (context)?”

### Eligibility criteria

This review included studies reporting on characteristics, implementation, or outcomes of an adult RRT, MET, or CCOT in low and middle-income countries (LIMCs) based on the World Bank Atlas 2021 classification.^( 18 )^ We included only original studies, such as randomized and non-randomized clinical trials, cohort studies, descriptive cross-sectional studies, and qualitative studies available as full-text or conference papers. Conference papers were also included in this review to account for underrepresentation of LMICs in peer-reviewed journals.^( 14 , 19 )^ We excluded research protocols, meta-analyses, and non-original articles, such as viewpoints and narrative reviews.

### Search strategy

We developed a comprehensive search strategy informed by a professional librarian to identify relevant articles with search strings for the two main concepts: RRT/MET/CCOT and LMICs. We searched the following databases: MEDLINE Ovid (1946 to August 23, 2024), Embase Ovid (1974 to 2024 August 23), and Emcare Ovid (1995 to 2024 Week 34) to retrieve published studies. Preliminary research was conducted to identify relevant papers - 12 were identified, which comprised the gold set of key references. The gold set was examined for relevant Medical Subject Headings (MeSH) terms and keywords/phrases in the title and abstract using Yale MeSH Analyzer for the two main concepts. The final MEDLINE search strategy is available in the Supplementary Material .

### Study selection and data extraction

The results were uploaded to the Rayyan® platform, a literature management software. After duplicates were removed, two researchers independently screened the titles and abstracts of the studies for the inclusion and exclusion criteria. A study was included if at least one researcher classified it as eligible. Then, two researchers read the full text or the conference abstract and determined its suitability for inclusion. In case of any discrepancies regarding the eligibility of specific studies, the reviewers decided through consensus.

We developed a standardized and pre-tested form in Microsoft Excel® to extract data from the included studies for evidence synthesis. Data extracted contained details including the study design, setting, composition of the RRT, presence of a CCOT, baseline period and period after implementation of RRT, reported outcomes, role of the RRT/MET, number of RRT/ MET calls, number of observed blue codes, ICU transfer, ICU length of stay (LOS), hospital LOS and hospital mortality. Code blue was defined as a hospital emergency code used to indicate a patient requiring immediate resuscitation, typically due to cardiac or respiratory arrest.^( 20 )^

### Risk of bias of individual studies

Considering the purpose of this study to map the available evidence regarding RRT/ MET/ CCOT in LMICs, we did not assess the risk of bias of included studies, as recommended by JBI for scoping reviews.^( 21 )^

### Data synthesis and analysis

Results were summarized using descriptive statistics, and study characteristics are reported as frequencies and percentages. We used the PAGER (acronym from Patterns, Advances, Gaps, Evidence for practice and Research recommendations) framework^( 22 )^ to synthesize the domains assessed in this review. We used R version 4.4.1 for graphical presentations with the packages ‘tidyverse’, ‘maps’, and ‘ggrepel’. We did not perform quantitative synthesis.

## RESULTS

### Literature search

**Figure 2:**
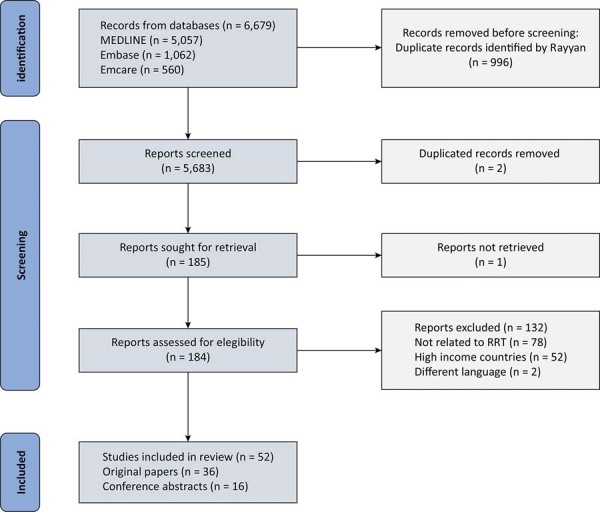
Flow chart showing the selection process. RRT - Rapid Response Teams.

We retrieved 6,679 records from the primary search, of which 996 were duplicate records and 5,683 records were screened for title and abstract. Subsequently, 185 studies were assessed for eligibility, and 52 were included. The main reasons for exclusion were a different theme from the main question (n = 78) and being settled in a high-income country (n = 52). Full-text studies accounted for 36 (69%) of the studies included, and 16 (31%) were conference abstracts ( Figure 2 ).

### Study characteristics

After reviewing the studies, we identified five main themes synthesized at the PAGER framework: study design, reported outcomes, impact on clinical outcomes, team composition, and additional roles ( Table 1 ).

**Table 1:** Main themes (patterns) identified through the PAGER framework for scoping reviews

Pattern	Advances	Gaps	Evidence for practice	Research recommendations
Study design	Observational studies and before -after studies evaluating RRTs	Lack of randomized controlled trials or robust multicentric before and after studies	Implementation of RRTs is feasible in various hospital settings	Conduct large-scale, well-designed clinical trials, especially in LMICs
Reported outcomes	Reported outcomes are heterogeneous, but usually include number of cardiac arrests and hospital mortality	Inconsistent outcome reporting and lack of standardized metrics.	Reporting of RRT dose, number of cardiac arrests to 1,000 admissions or discharges, and hospital mortality before and after RRT implementation	Encourage adoption of standardized outcome measures for RRT effectiveness evaluation
Impact on clinical outcomes	Demonstrated association with benefit in reducing intra-hospital cardiac arrest and, in some studies, mortality	Unclear causality between RRT implementation and improved outcomes due to study design limitations	Implementation of RRTs enhances early recognition of clinical deterioration and may reduce avoidable deaths.	New implementations should include robust impact evaluations. In LMIC settings, a large cluster randomized trial would be desired.
Team composition	Most teams in LMICs include physicians and nurses, with a few incorporating respiratory therapists	The ideal team composition and the role of intensivist-led teams in resource-constrained settings is not well described	Standardizing team composition based on available resources, with dedicated team members, may improve efficiency	Research on RRT models in different economic contexts, including the role of nurse-led RRTs and intensivist-led METs or hybrid teams in LMICs
Additional roles	Some studies reported roles of RRT beyond managing clinical instability and related consequences (as. code blue and mortality)	Impact of RRT on other roles, such as GOC discussions, ICU triage efficiency and others is not understood because of a lack of evidence	RRT may play a role in GOC/patient-centered discussions and healthcare professionals’ training	Studies on the role of RRT in education, GOC, research should be conducted

PAGER - pattern, advances, gaps, evidence for practice and research recommendations framework; RRT - Rapid Response Teams; LMIC - low and middle-income countries; MET - Medical Emergency Team; GOC - goals of care; ICU - intensive care unit.

### Study design

**Figure 3:**
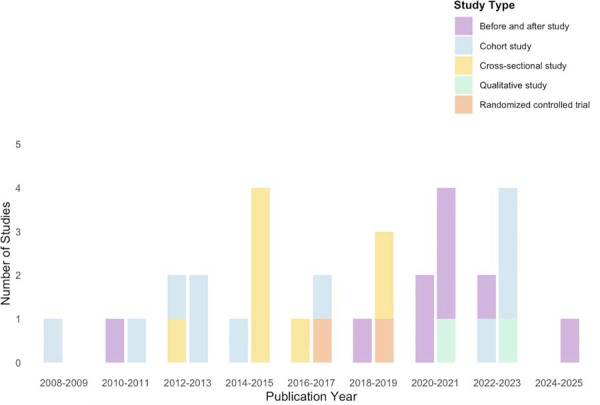
Number of studies by year and by type. The first bar represents full-text articles, and the second bar corresponds to conference abstracts.

The main characteristics of the selected studies are summarized in table 2 . Countries from South America, Asia, and Africa ( Figure 1S - Supplementary Material ) have published manuscripts about the concept. Most of the studies were from Brazil (51.2%), followed by India (19.2%) and Turkey (7.7%). The predominant study design was a before-and-after format (38.4%), followed by cohort studies (30.8%) and cross-sectional studies (21.1%). Three qualitative studies were addressing RRT members and their perceptions about the provided care and system organization.^( 23 - 25 )^Fifty-one studies were single-center and usually conducted in public (50%) or private hospitals (34.6%). The two earliest reports were published in 2009 as conference abstracts, and there was an increase in the number of published studies over the following years ( Figure 3 ).

**Table 2:** Summary of the characteristics of included studies

	n (%)
Study design	
Before and after study	20 (38.4)
Cohort study	16 (30.8)
Cross-sectional study	11 (21.1)
Qualitative study	3 (5.8)
Randomized controlled trial	2 (3.8)
Countries	
Brazil	27 (51.9)
India	10 (19.2)
Turkey	4 (7.7)
Iran	4 (7.7)
China	3 (5.8)
Other*	4 (7.7)
Single center	51 (98.1)
Type of hospital	
Public	26 (50)
Private	18 (34.6)
Not specified	8 (15.4)
Critical care outreach team	2 (2.8)
Reported outcomes	
Hospital mortality	26 (50)
Code blue	21 (40.4)
ICU admission	21 (40.4)
Hospital LOS	8 (15.4)
ICU LOS	6 (11.5)
Goals of care discussion	4 (7.7)

ICU - intensive care unit; LOS - length of stay. * Egypt, Tunisia, Pakistan, and Lebanon.

### Reported outcomes and impact on clinical outcomes

Table 3 summarizes the characteristics of all published studies and reported outcomes. The number of RRT calls in the before-and-after studies varied from 2.39^( 26 )^to 124^( 27 )^ per 1,000 admissions. Several studies described only the absolute number of RRT calls or number of assessed patients.^( 16 , 23 , 28 - 58 )^ Reported outcomes in the before-and-after studies also varied, with most studies focusing on mortality and code blue incidence. Twenty-six studies described hospital mortality,^( 26 - 28 , 30 - 32 , 34 - 36 , 42 - 44 , 47 , 48 , 50 , 52 , 57 , 59 - 67 )^ one study described 30-day survival^( 46 )^and another study also reported the hospitalization cost before and after RRT implementation.^( 48 )^

**Table 3:** Characteristics of the studies

Author	Country	Study design	Study objective	Team composition	Data available on RRT calls and/or sample size	Main findings	Other findings
Full-text							
Bergamasco et al.^( 16 )^	Brazil	Before and after study	To compare readmission rates in the ICU before and after RRT	ICU physician + physical therapist	ICU admissions: 380 (before RRT) *versus* 1,361 (after RRT)	ICU readmission: 35 (9.2%) *versus* 91 (6.7%), p = 0.093	ICU LOS: 3(1 - 5) *versus* 2 (1 - 6), p = 0.354; hospital LOS: 16 (10 - 26.5) *versus* 17 (9 - 31), p = 0.647
Jeddian et al.^( 23 )^	Iran	Qualitative study	To explore hospital staff perceptions of the perceived challenges and outcomes of a CCOT	ICU nurse	NA	Perceived challenges to CCOT: the hospital context and staff resistance to different nursing priorities, routines, and extra work. Positive outcomes: alleviating equipment shortages, improving nursing knowledge and patient care, and improving satisfaction	NA
Dias et al.^( 24 )^	Brazil	Cross-sectional study	To describe the construction of a questionnaire to assess the quality of care of RRT	NA	NA	At the end of the study, the questionnaire contained 37 statements, achieving a final compliance level higher than 80% in all concepts	NA
Yuan et al.^( 25 )^	China	Qualitative study	To conduct semi-structured interviews with non-nurse members of the RRT	NA	NA	Three themes were extracted: professional theoretical knowledge, professional practical skills, and personality traits	NA
Gong et al.^( 26 )^	China	Before and after study	To compare hospital outcomes before and after RRTs	ICU fellow + ICU nurse or respiratory therapist (as needed)	RRT calls: 834, 2.39/1,000 patients	Code blue: 301 *versus* 394, 0.23 *versus* 0.17/1,000 patient-days, p = 0.379; hospital mortality: 2.95 to 1.77/1,000 patient-days, p = 0.001; ICU admission: 2,141 *versus* 3,073/year, p = 0.002	NA
Viana et al.^( 27 )^	Brazil	Before and after study	To evaluate the impact of RRT implementation on the profile of IHCA	ICU physician	RRT calls: 124/1,000 admissions	Cardiac arrest: 4.2 *versus* 2.5/1,000 admissions, p < 0.001; hospital mortality after cardiac arrest: 86.9% *versus* 87.21%, p = 0.95	NA
Alves Silva et al.^( 28 )^	Brazil	Before and after study	To evaluate whether RRT triggered by the MEWS ≥ 4 in high-risk wards offering ED is associated with a decreased in in-hospital mortality	NA	Hospital admissions: 6,863 (before RRT) *versus* 6,844 (after RRT)	Hospital mortality: 534 (7.78%) *versus* 449 (6.46%), p = 0.003	Hospital LOS: 10.04 *versus* 9.82
Rocha et al.^( 29 )^	Brazil	Before and after study	To present the impact of the COVID-19 pandemic on a RRT operation	Physician with intensive care experience	RRT calls: 15,461, 30.6 (before COVID) *versus* 79.2 (after COVID)	ICU admission: 2.29% *versus* 6.23% (p < 0.001)	NA
Sabahi et al.^( 30 )^	Iran	Before and after study	To compare outcome measures before and after introducing RRT	Junior physician + Internal medicine physician (as needed)	Hospital admissions: 25,348 (before RRT) *versus* 28,024 (after RRT)	Code blue: 431 *versus* 349, p = 0.003; hospital mortality: 274 *versus* 231, p = 0.004	NA
Sedghiani et al.^( 31 )^	Tunisia	Cohort study	To describe characteristics and outcomes of MET calls	Emergency physician	RRT calls: 51	Hospital mortality: 12 (23%); ICU admission: 16 (31%)	NA
Taguti et al.^( 32 )^	Brazil	Cohort study	To describe data from clinical instability events in the adult inpatient units	ICU physician + physical therapist	RRT calls: 150 Patients: 104	Hospital mortality: 59 (56.7%); overall hospital mortality: 4.3%; ICU indication: 45 (43%)	NA
Boniatti et al.^( 33 )^	Brazil	Cohort study	To describe the reasons for MET activation and to verify the association of calling criteria with 30-day mortality	ICU physician	RRT calls: 1,051 Patients: 901	30-day mortality: 136 (35.7%); ICU admission: 489 (54%)	Code status changed to DNR after MET call: 30 (3.3%)
Boniatti et al.^( 34 )^	Brazil	Cohort study	To evaluate the frequency of RRT calls by the time of day and their association with in-hospital mortality	ICU physician	RRT calls: 9,220 Patients: 4,552	Hospital mortality: daytime call 614 (24.4%) *versus* nighttime call 467 (23.2%), p = 0.362; ICU admission: 552 (22%) *versus* 416 (20.7%), p = 0.307	Decrease in the number of calls during nursing handover periods: 7:00, 13:00, and 19:00; Increased mortality: 12:00 - 13:00 and 19:00 - 20:00
Tezcan et al.^( 35 )^	Turkey	Cohort study	To determine the predictors of in-hospital mortality for hospitalized patients	Resident physician + non-specified nurse	RRT calls: 545, 45/month	Hospital mortality: 189 (35.6%); ICU admission: 16 (31%)	NA
Duger et al.^( 36 )^	Turkey	Cohort study	To evaluate the MET and to determine the mean response time to calls, resuscitation success rate, and the rate of MET calls of clinics in the hospital	ICU physician + resident physician	Patients with IHCA: 132	Hospital mortality: 48 (36.4%)	NA
Chandrasegarane et al.^( 37 )^	India	Cohort study	To describe the characteristics of MET calls	NA	MET calls: 657	ICU admission: 225 (34.25%)	NA
Srivastava et al.^( 38 )^	India	Cross-sectional study	To identify the factors leading to overactivation and further improvement of RRT	Anesthesiologist + junior physician	RRT calls: 400	Code blue: 11 (2.7%)	NA
Mangal et al.^( 39 )^	India	Cross-sectional study	To evaluate the impact of RRT on patient outcome	ICU physician + ICU fellow + nursing supervisor	RRT calls: 50	Code blue: 2.44%; Mortality: 4.88%; ICU admission: 75.6%	ICU LOS: 2.55 days
Gülaçti et al.^( 40 )^	Turkey	Cross-sectional study	To evaluate the outcomes of in-hospital resuscitation after the introduction of code blue and MET	Anesthesiologist or Emergency physician (as needed) + non-specified nurse	Code blue calls: 264	Cardiac arrest: 186 (70%); ROSC: 109 (58%)	NA
Eroglu et al.^( 41 )^	Turkey	Cross-sectional study	To determine the cases of wrong blue codes and the reasons of misuse	Non-specified nurse + Emergency or ICU physician (as needed)	Code blue calls: 89	Cardiac arrest: 8 (9%)	NA
Santos et al.^( 42 )^	Brazil	Cross-sectional study	To analyze the characteristics of the activation of the yellow code and identify factors associated with adverse events after RRT call	NA	RRT calls: 107 Patients: 91	Code blue: 1(1.1%); ICU admission: 34(37.4%); Hospital mortality: 15 (16.5%)	NA
Yang et al.^( 43 )^	China	Cross-sectional study	To assess the impact of a RRS on the timely treatment of patients with serious adverse events	ICU and Emergency physician + non-specified nurse	RRT calls: 198; Patients: 208	Code blue: 86 (43.4%); hospital mortality: 15 (7.6%); ICU indication: 61 (30.8%)	NA
Jeddian et al.^( 44 )^	Iran	Randomized controlled trial	To explore the impact of a CCOT	ICU nurse	13 wards included in a cluster stepped-wedge trial: 7802 patients (before CCOT) *versus* 10,880 (after CCOT)	Hospital mortality: 370 (4.74%) *versus* 384 (3.53%), p = 0.913; CPR: 4.86% *versus* 3.61%, p = 0.999; ICU admission: 1.28% *versus* 1.23%, p = 0.644	Hospital LOS: 6(3 - 10) *versus* 4 (2 - 8), p = 0.971
YekeFallah et al.^( 45 )^	Iran	Randomized controlled trial	To determine the effect of RRT on the outcome of patient care	ICU nurse	Patients: 357 (no-RRT) *versus* 357 (RRT)	Code blue: 4 (1.1%) *versus* 0, p = 0.13; ICU admission: 24 (6.7%) *versus* 22 (6.2%), p = 0.76	NA
Sessim Filho et al.^( 48 )^	Brazil	Before and after study	To assess if RRT reduces in-hospital mortality and/or hospitalizations costs in a private general hospital in Brazil	NA	Patients: 529 (pre-RRT) *versus* 389 (after RRT)	Hospital mortality: 170 (30.41) *versus* 145 (24.74), p = 0.032	ICU LOS: 4 (2 - 7) *versus* 3(2 - 6), p = 0.113 Hospital LOS: 23 (12 - 40) *versus* 20 (11 - 36), p = 0.072 Hospitalization costs: $56,625.38 *versus* 46,272.37, p = 0.001
Yousaf et al.^( 59 )^	Pakistan	Before and after study	To compare hospital-wide code rates and mortality before and after RRT	ICU fellow + ICU nurse + respiratory therapist	RRTs calls: 796, 19.81/1,000 admissions	Code blue: 146 (0.369%) *versus* 148 (0.368%), p = 0.929; hospital mortality: 1,470 (3.725%) *versus* 1,529 (3,805%), p = 0.576	Code status changed to DNR after RRT call: 40 (5.03%)
Hosny et al.^( 60 )^	Egypt	Before and after study	To assess the impact of an RRT on the rates of inpatient mortality, CPR calls and unplanned ICU admission	ICU physician + non-specified physician or nursing supervisor (as needed)	Hospital admissions: 4,242 (before RRT) *versus* 2,338 (after RRT)	Code blue: 31 vs 4; 7.41 *versus* 1.77/1,000 discharges, p = 0.023; hospital mortality: 372 *versus* 105, 88.93 *versus* 46.44/1,000 discharges, p = 0.006; ICU admission: 5.98 *versus* 4.87/1000 discharges, p = 0.621	NA
Gonçales et al.^( 61 )^	Brazil	Before and after study	To evaluate the impact of RRT on the rate of IHCA and associated mortality	ICU physician	Hospital discharges: 40,033 (before RRT) *versus* 42,796 (after RRT)	Code blue: 3.54 *versus* 1.69/1,000 discharges, p < 0.001 ; hospital mortality: 16.27 *versus* 14.34/1,000 discharges, p = 0.029	NA
Mezzaroba et al.^( 62 )^	Brazil	Cohort study	To evaluate the implementation of a multidisciplinary RRT led by an ICU physician	ICU physician + physical therapist	RRT calls: 1,628, 49.12/1,000 admissions Patients: 1024	Code blue: 8.93/1,000 admissions; hospital mortality excluding code blue: 571 (67.7%)	Transition to palliative care: code yellow 5.3% and code blue 7.8%
Carvalho et al.^( 63 )^	Brazil	Cohort study	To evaluate the end-of-life care after limitations of medical treatment as defined by an RRT	ICU physician	RRT calls: 5396, 126/1,000 discharges Patients: 2,937 Patients with LOMT: 242	Hospital mortality: 204 (84.3%); overall hospital mortality: 3,468 (4.92%)	Palliative measures and LOMT: withhold mechanical ventilation 72 (29.8%), psychological support 32 (13.2%), withhold dialysis 24 (9.95)
Arora et al.^( 64 )^	India	Cohort study	To determine the patient characteristics, causes, and outcomes of RRT	ICU physician	RRT calls: 135, 9.8/1,000 admissions	Hospital mortality: 50 (37%)	ICU LOS: 7 (± 7.6) Hospital LOS: 8.6 (± 12.8)
Almeida et al.^( 67 )^	Brazil	Cross-sectional study	To describe the implementation of a RRT indicating relevant issues for other initiatives in similar contexts	ICU physician + ICU nurse + Internal medicine physician	RRT calls: 9.2/1,000 admissions	Code blue 32 (4.9%); overall hospital mortality 3.5%; ICU indication 158 (27.1%)	RRT calls for patients in palliative care: 67 (10.3%)
Menon et al.^( 68 )^	India	Before and after study	To implement MET and evaluate its effectiveness in reducing IHCA and mortality	Internal medicine physician + resident physician + nursing supervisor + non-specified nurse + respiratory therapist	RRT calls: 386, 17/1,000 patients	Code blue: 6.9 *versus* 2.6/1,000 admissions, p < 0.001; mortality after code blue: 4.96 *versus* 1.75/1,000 admissions, p = 0.601	NA
Boniatti et al.^( 69 )^	Brazil	Cohort study	To determine whether there was an association between delayed MET calls and mortality	ICU physician	RRT calls: 1481, 40/1000 admissions Patients: 1418	30-day mortality: timely MET activation 378 (41.9%) *versus* delayed MET activation: 152 (61.8%); ICU admission: timely 333 (35.9%) *versus* delayed 155 (63%)	DNR: timely 104 (11.5%) *versus* delayed 43 (17.5%)
Queiroz et al.^( 70 )^	Brazil	Cross-sectional study	To verify the perception of nurses of the quality of the RRT in the structure, process, and outcome dimensions	NA	NA	Satisfactory positive index was identified in 25 of the 37 items analyzed; there was a discrepancy in the perception of professionals with different lengths of time in the institution about the request for RRT (p = 0.03)	NA
Jamous et al.^( 71 )^	Lebanon	Before and after study	To investigate the effectiveness of implementing an RRT on patient outcomes	ICU nurse	Code team calls: 20.45%/1,000 discharges *versus* RRT calls 33.6%/1,000 discharges	Survival per discharge: 25% *versus* 50%/1000 discharges	NA
Dias et al.^( 72 )^	Brazil	Qualitative study	To analyze emergency care from the perspective of RRT professionals when faced with positive and negative critical incidents	NA	NA	Clusters for 89 critical incidents were obtained; 66 of them were considered positive, whereas 23 were negative. The situations associated to the services provided were discriminated in recognition of clinical deterioration; RRT activation; and time until RRT arrival	NA
Conference abstract							
Hajjar et al.^( 46 )^	Brazil	Before and after study	To evaluate the effectiveness of a multidisciplinary MET on CPR outcomes in cancer patients	ICU nurse + non-specified physician + respiratory therapist	Patients with IHCA: 82	30-day survival: 3.7% *versus* 14% (p = 0.017)	NA
Georgeto et al.^( 47 )^	Brazil	Before and after study	To compare clinical outcomes of critically ill patients treated in general hospital wards after the implementation of RRT	ICU physician	Patients: 699 (pre RRT) *versus* 889 (after RRT)	Hospital mortality: 124 (17.74%) *versus* 190 (21.37%); ICU admission: 418 (59.8%) *versus* 406 (45.67%), p < 0.001	Code status changed after RRT call: 34 (3.82%)
Flato et al.^( 49 )^	Brazil	Before and after study	To evaluate the result of RRT in reducing in-hospital morbidity and mortality	ICU physician	NA	Code blue: 1/month *versus* 5/month, p < 0.001	NA
Oliveira et al.^( 50 )^	Brazil	Before and after study	To determine the effect of a RRT on IHCA, ICU admissions, and ICU and hospital mortality	NA	Patients before RRT: 610 RRT calls: 276	IHCA: 14 *versus* 8, p = 0.87; hospital mortality: 62 *versus* 91%, p = 0.87	NA
Bolinedi et al.^( 51 )^	India	Before and after study	To evaluate the impact of the MET call system on the number of code blue events clinical outcomes	NA	MET calls: 91 (before MET reinforcement policy) *versus* 135 (after MET reinforcement)	Code blue: 53 *versus* 38	ROSC 66% *versus* 64%
Pardini et al.^( 52 )^	Brazil	Cohort study	To describe the various criteria for calling the RRT for patients who developed sepsis, initial treatment before transfer to the ICU or step-down unit and outcomes	NA	Patients: 65	Hospital mortality: 13 (20%)	ICU LOS: 3 (1 - 6) Hospital LOS: 26 (10 - 71)
Tiwari et al.^( 53 )^	India	Cohort study	To determine whether MET implementation improved outcomes	NA	Patients admitted to ICU: 203, 101 (no-MET) *versus* 102 (MET activation)	Code blue: 2 (no-MET) *versus* 8(MET), p = 0.05; 72 hours mortality: 16 (no-MET), *versus* 25.5% (MET), p > 0.05	NA
Palomba et al.^( 54 )^	Brazil	Cohort study	To determine what factors are associated with hospital mortality for patients seen by the RRT	NA	Patients: 513 (RRT call < 48 hours from hospital admission) *versus* 537 (RRT call > 48 hours)	ICU admission: 460 (43.7%)	Late RRT associated with mortality: (OR 2.73, 95%CI 1.01 - 1.04)
Grion et al.^( 55 )^	Brazil	Cohort study	To evaluate initial performance of a RRT	NA	RRT calls: 165	Code blue: 10	NA
Galindo et al.^( 56 )^	Brazil	Cohort study	To describe clinical characteristics, resources, main outcomes, and to address predictors of hospital mortality among patients admitted to the ICU/SDU after RRT activation	NA	Patients admitted to ICU: 3,841 (after RRT call)	Hospital survival: 3,165 (82.4%) *versus* 676 (17.6%)	Non-survivors were older, had higher SAPS III and longer hospital LOS before ICU
Kapadia et al.^( 57 )^	India	Cross-sectional study	To describe the characteristics of RRT	NA	Patients: 94	Code blue: 1 (1.1%); hospital mortality: 38 (40%); ICU admission: 69 (73%)	NA
Bonatto et al^( 58 )^	Brazil	Cross-sectional study	To compare the outcome of the patients cared by ED or ICU RRT	ICU physician	RRT calls: 122, 25 (RRT with ED physician) *versus* 87 (RRT with ICU physician)	Hospital discharge: 20 (80%) *versus* 71 (81.6%); ICU admission: 15 (60%) *versus* 53 (60.9%)	NA
Sudarshan Reddy et al.^( 65 )^	India	Before and after study	To assess the impact of MET on patient outcomes	NA	RRT calls: 889	Code blue: 0.82 (pre-MET) *versus* 2.4/1,000 admissions (MET), p < 0.001; hospital mortality: 15.6 *versus* 16.9/1,000 admissions, p = 0.43; ICU admission after MET: 222 (24.7%)	ICU LOS: 4 (3 - 8) *versus* 4 (1 - 8), p = 0.7
Narakurthi et al.^( 66 )^	India	Before and after study	To determine the impact of RRT on outcomes in a hospital	ICU physician + ICU nurse	Hospital admissions: 5,522 (pre-RRT) *versus* 6,956 (after RRT) RRT calls: 83, 11.9/1,000 admissions	Code blue: 22 *versus* 25; 1.16 *versus* 1.03/1,000 patient-days; hospital mortality: 21 *versus* 43; 1.84 *versus* 1.78/1,000 patient-days	Hospital LOS: 0.22 *versus* 0.14/1,000 patient-days
Viana et al^( 73 )^	Brazil	Before and after study	To evaluate changes in the characteristics of IHCA after RRT	NA	Patients with IHCA: 122 (pre-RRT) *versus* 188 (after RRT); RRT calls: 124/1,000 admissions	Code blue: 4.2/1,000 admissions (pre-RRT) *versus* 2.5/1,000 (after RRT) Mortality: 75 (87.2%) *versus* 153(86.9%), p = 0.95	ROSC: 42 (33.9%) *versus* 81 (43.1%), p = 0.103

ICU - intensive care unit; RRT- Rapid Response Teams; LOS - length-of-stay; CCOT - Critical care outreach team; NA - not available; IHCA - in-hospital cardiac arrest; MEWS - Modified Early Warning Score; ED - Emergency Department; MET Medical Emergency Team; ROSC - return of spontaneous circulation; RRS - Rapid Response System; DNR - do-not-resuscitate; LOMT - limitations of medical treatment; CPR - cardiopulmonary resuscitation; SDU - step-down unit; SAPS - Simplified Acute Physiology Score.

Comparative incidence of code blues before and after RRT was reported in 12 studies using different metrics.^( 28 , 29 , 32 , 52 , 54 , 62 - 64 , 66 , 68 - 70 )^ The full-text studies that described the rate of code blue per 1,000 patient admissions or discharges identified 3.54 to 7.41 code blues per 1,000 patient admissions or discharges before RRT implementation compared to 1.69 to 2.6 code blues per 1,000 patient admissions or discharges after RRT^( 27 , 60 , 61 , 68 )^ ( Table 1S - Supplementary Material ). Four studies reported an active role of RRT in goals of care discussion, with a reported change of code status to do not resuscitate after RRT evaluation.^( 33 , 47 , 59 , 63 )^ None of the studies^( 16 , 23 - 73 )^ reported the use of an automated electronic system for RRT activation. One study reported that delayed medical emergency calls were associated with increased mortality (61.8% *versus* 41.9%) compared to timely MET activation.^( 69 )^

### Team composition

Thirty-four studies^( 16 , 23 , 26 , 27 , 29 - 36 , 38 - 41 , 43 - 47 , 49 , 58 - 64 , 66 - 69 , 71 )^ provided data on the composition of RRT. Four studies^( 23 , 44 , 46 , 71 )^ reported an exclusive nurse-led team and ICU nurses were always present in 23% of the RRTs. In 56% of the teams, an ICU physician was involved in RRT calls, and less frequently, emergency physicians (6%), internal medicine physicians (6%), and anesthesiologists (3%) were present. The mandatory participation of ICU fellows in RRT was described in 9% of the studies. A physical or respiratory therapist was always involved in 18% of the studies and sometimes involved in 3% ( Table 2S - Supplementary Material ).

### Additional roles

There was no clear description of additional roles of RRT beyond those reported above.

## DISCUSSION

We found in this scoping review that the published literature regarding RRT on LMICs is scarce and limited to a few countries -, even though there is a trend towards increasing the number of publications in recent years. We observed methodological heterogeneity in study design and reported outcomes, often presentation of incomplete data, which limits the interpretation of the actual impact of RRT in these settings. In addition, team composition was not reported in 34% of the studies. These findings highlight that a significant knowledge gap regarding RRTs in LMICs remains despite ongoing efforts.

### Uptake in low and middle-income countries

One of the first reports of a MET was by Lee et al. in 1995 from a hospital in Australia.^( 74 )^ Rapid Response Teams spread in the following years, and they were present in over 60% of ICU-equipped Australian hospitals in 2005,^( 75 )^ even though the only multicentric randomized controlled trial evaluating the benefit of this intervention was neutral.^( 76 )^ In this review, the first study we retrieved from LMICs was from 2009, and most of the studies are from the last 10 years, evincing an important time difference until the beginning of the implementation of RRTs in LMICs. We found studies particularly from middle-income countries; low-income countries were further underrepresented (there were only two studies from the African continent: one from Egypt and another from Tunisia, both lower-middle-income countries). This highlights an important gap to be addressed, as critically ill patients in the African continent are frequently treated in general wards.^( 77 )^In addition, the preferred study design was a before-and-after format or cohorts, with no standardized metrics for evaluation.

Another topic increasingly explored in recent years is how the activation flow of RRTs can be optimized to improve patient outcomes. Electronic systems based on early warning scores (EWS) or other clinical triggers automate RRT activation and enable early identification of deterioration.^( 78 )^ These systems aim to overcome delays in activation, which have consistently been associated with increased mortality in both high-income and low- and middle-income settings.^( 69 , 79 )^

### Reported outcomes

Reported outcomes varied in the included studies, and not all of them contained information about the number of RRT calls, cardiac arrests, and mortality before and after implementation of this system. The number of RRT calls per 1,000 patient admissions or discharges, known as “RRT dose”, is directly related to improvement of patient outcomes and is a necessary process measure to evaluate the implementation of RRT.^( 80 )^ The minority of included studies reported RRT calls using the proper metric, and many of them showed an activation rate smaller than the 20 - 40 per 1,000 admissions or discharges recommended in the literature.^( 1 )^ Even so, most of the studies showed a reduction in the primary outcome evaluated, whether it was the number of code blues, mortality or ICU admission. The four before-and-after studies presented as full-text all reported a decrease in code blue after RRT implementation to less than 2.6 per 1,000 admissions or discharges, consistent with the benefit observed in HICs.^( 20 )^ Considering the lack of robust reporting of RRT dose or outcome measurement, the interpretation and generalization of these results are limited.

### Team composition

Ideal RRT composition is uncertain in HIC: composition of MET in Australia includes a physician as team leader, usually an ICU fellow, and a skilled nurse as a team member;^( 81 )^ in the United States, over 70% of hospitals present a nurse-led RRT; in England, CCOT in a nurse-led service, with frequent consultations from ICU physicians.^( 82 )^Our review showed a higher prevalence of physicians in RRTs, including physician-exclusive teams, with a lesser presence of a dedicated nurse. Compared to HIC, LMICs frequently face worse nurse-to-patient ratios, longer working hours, lower remuneration, and reduced nurse autonomy related to work overload.^( 83 , 84 )^ These factors may contribute to the predominance of physician-centered models in RRTs across many LMICs.

### Other roles of Rapid Response Teams

Considering that the initial focus of RRT implementation is to reduce intra-hospital cardiac arrest, it is not surprising that other roles, such as goals of care discussion, educative initiatives, and technical support for non-specialists for reducing moral distress, were scarcely reported. These approaches were only later described as benefits of RRT in HICs.^( 10 , 11 )^ However, especially in resource-constrained settings, these initiatives could also optimize resource utilization by prioritizing ICU transfers for patients who would most likely benefit from scarce ICU beds, and prompt discussions based on other patient-centered outcomes.

### Implications

Implementation of RRT is feasible and strategic in managing clinical deterioration outside of the ICU. Current evidence in LMICs lacks consistency, rigorous study design and standardized evaluation of outcomes. To properly evaluate the impact of RRS, studies should consistently report a core set of data elements that include: RRT dose; the rate of in-hospital cardiac arrests per 1,000 admissions; overall hospital mortality; unexpected ICU admissions; hospital characteristics; how the response team is structured; how activations are triggered; whether staff are trained using standardized tools or protocols; for each event, patient demographics, the reasons for activation, timing, clinical interventions, patient destination, and any changes in code status (such as new do-not-resuscitate orders).^( 12 , 85 )^Adapting team composition to available resources is also important, since there is no consensus for optimal team composition.

Furthermore, automated RRT activation systems and the increasing use of artificial intelligence to recognize triggers of clinical deterioration should be further explored as promising strategies to enhance the effectiveness of rapid response interventions, and their cost-effectiveness needs to be evaluated in LMICs. There is an urgent need for well-designed studies, ideally hybrid effectiveness-implementation^( 86 )^ studies, that integrate the assessment of digital alert systems, RRT structure, and training in LMICs and that identify and address barriers to implementation, given the evidence base in the overall literature supporting RRS. Future research should also explore the expanding roles of RRTs, including goals of care discussions, ICU triage, and the development of communication skills and educational strategies.

### Strengths and limitations

We chose a scoping review model because we anticipated that evidence on RRT at LMICs would be heterogeneous, precluding meta-analysis to analyze the findings. In addition, our main goals were to identify the available evidence in the literature, identify equity-related challenges, knowledge gaps, and research opportunities.^( 87 )^

There are several limitations to our scoping review, inherent to the characteristics of the published literature. Variability in design and reported outcomes of the studies implies difficulty in extracting data and gathering information for a global interpretation. Although using conference abstracts aims to expand the search considering lesser publication of LMICs in high-impact journals, it also adds a layer of incomplete data because only main results are usually reported, and secondary outcomes that may have been relevant are omitted. Finally, we limited our research to three databases and did not include other sources, such as dissertations, government reports, and further gray literature, which may have added information. Nonetheless, this choice stresses the existing knowledge gap in the most common and accessible sources of medical information.

## CONCLUSION

Our findings demonstrate a lagged published literature describing the implementation of Rapid Response Teams in low- and middle-income countries, with inconsistent team composition, inconsistent outcome reporting, and ultimately unclear best practices to be followed. Further studies addressing the best team composition, automated electronic system activation, and additional desired roles of Rapid Response Teams with proper impact and cost-effectiveness evaluation are needed to advance the concept of low and middle-income countries, where it could have an even greater impact on clinical outcomes.

## SUPPLEMENTARY MATERIAL

SUPPLEMENTARY MATERIAL
