# Supplementary material for: Rapid Response Teams in low and middle-income countries: a scoping review
Source: Crit Care Sci. 2025 Oct 1;37:e20250155. doi: 10.62675/2965-2774.20250155 (PMC12614955; doi:10.62675/2965-2774.20250155)
Supplement: SUPPLEMENTARY MATERIAL [file 2965-2774-ccsci-37-e20250155-suppl01.pdf]

Critical Care Science
https://doi.org/10.62675/2965-2774.20250155

SUPPLEMENTARY MATERIAL

Rapid Response Teams in low and middle-income countries: a scoping review

Larissa Bianchini[1,2,3], Luiz Marcelo Almeida de Araújo[2], Daryl Jones[4], Bruno Adler Maccagnan Pinheiro Besen[1,2,5]

SEARCH STRATEGY

Ovid MEDLINE(R) ALL <1946 to August 29, 2024>

#	Query	Results from 29 Aug 2024
1	patient care team/ or hospital rapid response team/	71,544
2	patient care team*.ti,ab,kf.	1,023
3	rapid response team*.ti,ab,kf.	1,324
4	critical care outreach.ti,ab,kf.	184
5	medical emergency team*.ti,ab,kf.	797
6	code team*.ti,ab,kf.	125
7	cardiac crash team*.ti,ab,kf.	1
8	1 or 2 or 3 or 4 or 5 or 6 or 7	73,347
9	Developing Countries/	82,697
10	low income countr*.ti,ab,kf.	11,069
11	middle income countr*.ti,ab,kf.	41,100
12	lower middle income countr*.ti,ab,kf.	2,164
13	upper middle income countr*.ti,ab,kf.	1,026
14	LMIC*.ti,ab,kf.	12,901
15	(developing countr* or developing nation*).ti,ab,kf.	109,599
16	(least developed countr* or least developed nation*).ti,ab,kf.	376
17	(less developed countr* or less developed nation*).ti,ab,kf.	1,650
18	(third world nation* or third word countr*).ti,ab,kf.	57
19	(under developed nation* or under developed countr*).ti,ab,kf.	166
20	(Association between time of day for rapid response team activation and mortality).m_titl.	1
21	(Evaluation of a critical care outreach service in a middle-income country: A stepped wedge cluster randomized trial and nested qualitative study).m_titl.	1
22	Implementation of a rapid response team in a large nonprofit Brazilian hospital: improving the quality of emergency care through Plan-Do-Study-Act.mp. [mp=title, book title, abstract, original title, name of substance word, subject heading word, floating sub-heading word, keyword heading word, organism supplementary concept word, protocol supplementary concept word, rare disease supplementary concept word, unique identifier, synonyms, population supplementary concept word, anatomy supplementary concept word]	1
23	Implementing a Rapid Response Team: A Quality Improvement Project in a Low- to Middle-Income Country.mp. [mp=title, book title, abstract, original title, name of substance word, subject heading word, floating sub-heading word, keyword heading word, organism supplementary concept word, protocol supplementary concept word, rare disease supplementary concept word, unique identifier, synonyms, population supplementary concept word, anatomy supplementary concept word]	1

Continue...

This is an open access article under the CC BY license (https://creativecommons.org/licenses/by/4.0/).

...continuation

24	Dealing with the impact of the COVID-19 pandemic on a rapid response team operation in Brazil: Quality in practice.mp. [mp=title, book title, abstract, original title, name of substance word, subject heading word, floating sub-heading word, keyword heading word, organism supplementary concept word, protocol supplementary concept word, rare disease supplementary concept word, unique identifier, synonyms, population supplementary concept word, anatomy supplementary concept word]	1
25	Analysis of readmission rates to the intensive care unit after implementation of a rapid response team in a University Hospital. mp. [mp=title, book title, abstract, original title, name of substance word, subject heading word, floating sub-heading word, keyword heading word, organism supplementary concept word, protocol supplementary concept word, rare disease supplementary concept word, unique identifier, synonyms, population supplementary concept word, anatomy supplementary concept word]	1
26	Nurses' perception of the quality of the Rapid Response Team.m_titl.	1
27	"A quality improvement initiative to reduce out-of-icu cardiopulmonary arrests in a tertiary care hospital in india: A 2- year learning experience".m_titl.	1
28	"A rapid response team is associated with reduced overall hospital mortality in a Chinese tertiary hospital: a 9-year cohort study.".m_titl.	1
29	(Comparison of Hospital-Wide Code Rates and Mortality Before and After the Implementation of a Rapid Response Team).m_titl.	1
30	Clinical impact of implementing a rapid-response team based on the Modified Early Warning Score in wards that offer emergency department support.m_titl.	1
31	(Efficacy of a rapid response team on reducing the incidence and mortality of unexpected cardiac arrests).m_titl.	1
32	"low to middle income countr*".ti,ab,kf.	31,255
33	Afghanistan/ or Burkina Faso/ or Burundi/ or Central African Republic/ or Chad/ or Congo/ or Eritrea/ or Ethiopia/ or Gambia/ or Guinea/ or Korea/ or Liberia/ or Madagascar/ or Malawi/ or Mali/ or Mozambique/ or Niger/ or Rwanda/ or Sierra Leone/ or exp Somalia/ or South Sudan/ or Sudan/ or Syrian Arab Republic/ or Togo/ or Uganda/ or Yemen/ or Angola/ or Bangladesh/ or Benin/ or Bhutan/ or Bolivia/ or cabo Verde/ or Cambodia/ or Cameroon/ or Comoros/ or Cote d'Ivoire/ or Djibouti/ or Egypt/ or Eswatini/ or Ghana/ or Haiti/ or Honduras/ or India/ or Jordan/ or Kenya/ or Kiribati/ or Kyrgyzstan/ or Laos/ or Lebanon/ or Lesotho/ or Mauritania/ or Micronesia/ or Morocco/ or Myanmar/ or Nepal/ or Nicaragua/ or Nigeria/ or Pakistan/ or Papua New Guinea/ or Philippines/ or Samoa/ or "sao tome and principe"/ or Senegal/ or Solomon Islands/ or Sri Lanka/ or Tajikistan/ or Tanzania/ or Timor-Leste/ or Tunisia/ or Uzbekistan/ or Vanuatu/ or Vietnam/ or Zambia/ or Zimbabwe/ or Albania/ or Algeria/ or Argentina/ or Armenia/ or Azerbaijan/ or Belarus/ or Belize/ or "Bosnia and Herzegovina"/ or Botswana/ or Cuba/ or China/ or Brazil/ or Colombia/ or Costa Rica/ or Dominica/ or Ecuador/ or El Salvador/ or Fiji/ or Gabon/ or Georgia/ or Grenada/ or Guatemala/ or Indonesia/ or Iran/ or Iraq/ or Jamaica/ or Kazakhstan/ or Kosovo/ or Libya/ or Malaysia/ or Maldives/ or Mauritius/ or Mexico/ or Moldova/ or Mongolia/ or Montenegro/ or Namibia/ or "Republic of North Macedonia"/ or Paraguay/ or Peru/ or Serbia/ or South Africa/ or Suriname/ or Thailand/ or Tonga/ or Turkey/ or Turkmenistan/ or Tuvalu/ or Ukraine/	1,228,537
34	(Afghanistan or Afghani or Afghan or "Burkina Faso" or Burkinabe or Burundi* or "Central African Republic" or Chad or Tchad or Chadian or Congo or Congolese or Eritrea* or Ethiopia* or Gambia or gambian or equatorial or guinea or "Guinea-Bissau" or North Korea* or Liberia* or Madagascar or malagasy or Malawi* or Mali or Malian or Mozambique or Mozambican or Niger or Nigerien* or Rwanda* or "Sierra Leone" or Somalia* or South Sudan or Sudan or Sudanese or Syrian or Syria or Togo or Togolese or Uganda* or Yemen* or Angola* or Bangladesh* or Benin or beninese or Bhutan or Bhutanese or Bolivia* or Cabo Verde or cape verde or Cambodia* or Cameroon or Cameroonian or Comoros or Comoran or cote d'Ivoire or Ivorian or Djibouti or Egypt or egyptian or Eswatini or swazi or Ghana* or Haiti or Haitian or Hondura* or India or "Indian not American" or Jordan or Jordanian or Kenya* or Kiribati or "Kyrgyz Republic" or Kyrgyzstan or Laos or Laotian or Lebanon or "Lebanese republic" or Lebanese or Lesotho or Basotho or Mosotho or Mauritania* or Micronesia* or Morocco or moroccan or Myanmar or Burmese or myanmarese or Nepal or Nepalese or Nicaragua* or Nigeria* or Pakistan* or "Papua New Guinea" or Philippines or Filipino* or Samoa or "Sao Tome and Principe" or "San Tomean" or Senegal* or "Solomon Island" or Sri Lanka* or Tajikistan or tajik or Tadzhik or Tanzania* or "Timor Leste" or Timorese or Tunisia* or Uzbekistan or uzbeki or Vanuatu or Vietnam* or "West Bank" or Gaza or Zambia* or Zimbabwe* or Albania* or Algeria* or Argentina or argentine or Armenia* or Azerbaijan or Azerbaijanis or azeris or Belarus or Belize or "Bosnia and Herzegovina" or Bosnian or Botswana or Brazil or Brazilian or China or Chinese or Colombia* or "Costa Rica" or Cuba* or Dominica* or Ecuador or "El Salvador" or Fiji or Gabon or Georgia* or Grenada or Guatemala or Indonesia* or Iran or iranian or Iraq or Jamaica or Kazakhstan or Kosovo or Kosovar or Kosovan or Libya* or Malaysia* or Maldives or "Marshall Islands" or Mauritius or Mexico or Mexican or Moldova or Mongolia* or Montenegro or Namibia* or Macedonia* or Paraguay or Peru or Serbia* or South Africa or "St. Lucia" or "St. Vincent and the Grenadines" or Suriname or Thailand or thai or Tonga or turkey or turc or Turkmenistan or Tuvalu or Ukraine).ti,ab,cp,kf,in.	7,958,818
35	9 or 10 or 11 or 12 or 13 or 14 or 15 or 16 or 17 or 18 or 19 or 32 or 33 or 34	8,124,490
36	8 and 35	5,057

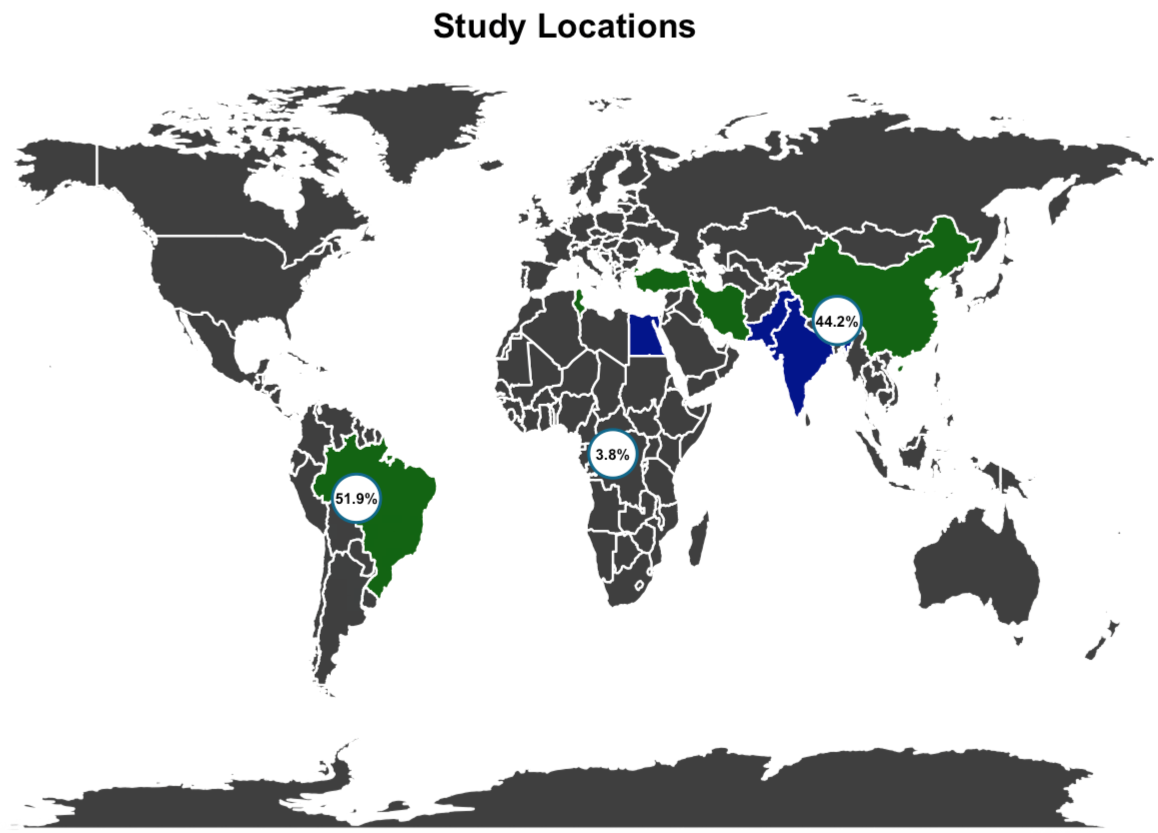

Figure 1S - Geographic location of included studies.

Table 1S - Summary of full-text before-and-after studies evaluating Rapid Response Teams

Author, year	Nature of RRT	Rates before RRT		Rates after RRT	
		Code blue incidence	Mortality	Code blue incidence	Mortality
Viana et al.[27] 2021	MET	4.2/1,000 admissions	IHCA mortality: 87.2%	2.5/1,000 admissions	IHCA mortality: 86.9%
Hosny et al.[60] 2024	MET	7.41/1,000 discharges	Overall hospital mortality: 88.93/1,000 discharges	1.77/1,000 discharges	Overall hospital mortality: 46.44/1,000 discharges
Gonçales et al.[61] 2012	MET	3.54/1,000 discharges	Code blue mortality: 2.33/1,000 discharges	1.69/1,000 discharges	Code blue mortality: 0.78/1,000 discharges
Menon et al.[68] 2018	MET	6.9/1,000 admissions	Code blue mortality: 4.93/1,000 admissions	2.6/1,000 admissions	Code blue mortality: 1.75/1,000 admissions

RRT - Rapid Response Team; MET - Medical Emergency Team; IHCA - intra-hospital cardiac arrest.

Table 2S - Summary of composition of Rapid Response Teams (n = 34)

**Personnel**	**Always involved**	**Sometimes involved**
Medical staff	28 (82.3)	
ICU fellow	3 (8.8)	
ICU physician	19 (55.9)	1 (3)
Resident physician	3 (8.8)	
Anesthesiologist	1 (2.9)	1 (3)
Internal medicine physician	2 (5.8)	1 (3)
Non-specified physician	4 (11.8)	1 (3)
Emergency physician	2 (5.8)	2 (6)
Nursing staff	14 (41.2)	
ICU nurse	8 (23.5)	1 (3)
Nursing supervisor	2 (5.8)	1 (3)
Non-specified nurse	5 (14.7)	
Physical or respiratory therapist	6 (17.8)	1 (3)

ICU - intensive care unit. Results are expressed as n (%).
